# Notes on the Peculiarities of Surgical Practice in the London Hospitals

**Published:** 1886-12-20

**Authors:** Charles N. Dixon Jones

**Affiliations:** Surgeon to Woman’s Hospital, Brooklyn, N. Y.; Fellow New York Academy of Medicine, etc.


### Southern Medical Record.

A MONTHLY JOURNAL OF PRACTICAL MEDICINE.

☞All Communications must be addressed to R. C. WORD, M. D., Managing Editor.

"*Quicquid Præcipies Esto Brevis.*"

VOL. XVI. ATLANTA, GA., DECEMBER 20, 1886. NO. 12

### ORIGINAL AND SELECTED ARTICLES.

### NOTES ON THE PECULIARITIES OF SURGICAL PRACTICE IN THE LONDON HOSPITALS.

BY CHARLES N. DIXON JONES, B. S. M. D.,
Surgeon to Woman's Hospital, Brooklyn, N. Y; Fellow New York Academy of Medicine, etc.

*To the Editor of the Southern Medical Record:*

During my stay in London I have seen many cases and peculiarities of practice which are of especial interest to me as a student of medicine in America. Hoping they may prove of similar interest to your readers I will attempt to describe some of the more important cases which have appeared at the clinics of the different London hospitals.

London possesses no less than twelve regular medical schools, one being devoted to the education of women, each of which prepare students for entering the ranks of the medical profession. Connected with each of these schools there is a fully-appointed hospital, thus affording their students the best possible opportunities for clinical study. In addition to these there are about nineteen important special hospitals, not directly connected with any school, yet affording students opportunities for study in special branches. It is a source of regret to students that there are so many distinct schools distributed in different portions of the metropolis, each of which presents attractions and advantages which claim his attention. It would be desirable to have the distinguished instructors centered at one or two institutions, as we find it on the Continent, or even in our own country, so that the great body of students could have the benefit of that teaching which is now restricted to those few who constitute the pupils of the several schools to which they are attached. Notwithstanding this fact, it must be conceded that there are certain advantages connected with the arrangement as it now exists. On account of their great number, the several schools have a comparative small number of students, thus affording the instructors the opportunity to become more intimately acquainted with the students, while on the other hand they derive the benefit of personal instruction. Every student may also have the opportunity of holding the position of clinical assistant, post mortem clerk and surgical dresser, certificates of having held these positions being required in order to obtain a degree. The student is also afforded the opportunity of seeing the different operative procedures as practiced at the different hospitals, as well as learning the conflicting opinions and various views as held by men of eminence at the different schools, all tending to endow him with a true liberality of spirit.

Homœopathy maintains but an embryonic existence in England, the regular school being so fairly established this later scion has little opportunity of taking root and assuming a flourishing condition. The London School of Homœopathy has been recently established in connection with the London Homœopathic Hospital, where any one who desires may obtain acquaintance with the principles and practice of the system known as "Homœopathy." They have lectures on materia medica and therapeutris, and the principles and practice of medicine, clinical instruction on these subjects also being given in the hospital. In the circular is announced that "the London School of Homœopathy should supply only that knowledge which cannot be acquired at any other hospital or medical school in Great Britain, viz.: instruction in the homœopathic materia medica and therapeutics, in the principles and practice of homœopathic medicine, and its application in clinical medicine, within the wards of our hospitals." This instruction is available to all students and members of the profession.

### MR. LISTER AT KING'S HOSPITAL — I. HOSPITAL.

A case was presented at King's College Hospital which afforded Mr. Lister an opportunity "to illustrate the progress that surgery is making in our period," (I quote his words from notes taken at the time) as well as the importance of clinical teaching in general. The patient, a man aged forty-five years, met with an accident five months ago. As he lies on the table you observe that the two anterior superior spinous processes of the ilium are on the same level ; there is no obliquity of the pelvis, and yet one limb is fully one inch or more shorter than the other. The thigh bone is not continuous. This might be due to different causes, to an accident, or softening of the bone and the growth of a tumor. But on looking over his history we discover that five months ago his foot was caught under a boiler and so rolled and twisted as to cause fracture of the thigh. The limb was put in splints, but at the end of six weeks no union had taken place. At the end of that time we should expect union. Why union has not taken place it is impossible to say ; from the obliquity of the fragments we would be led to suspect that muscle is interposed between the fragments. On measurement from the anterior superior spine of the ilium to the external malleolus, the right leg was found to measure $34\frac{1}{2}$ while the left measured but $31\frac{5}{8}$. In measuring the limb Mr. Lister does not place the tape on the limb, but the ends of the tape are held opposite to the points from which the measurement is made in the same vertical plane; in this way the errors due to the irregularities of the surface of the limb are avoided.

*As to the treatment.*—The tendencies are, when a bone is broken, for new material to be formed for its repair, and this new material partakes more or less of the nature of the injured tissue. How long would you expect this tendency to unite to continue? Bones differ in the rapidity with which they unite, all reparative processes are more rapid in the upper extremity. At the end of six weeks you would expect union. If union does not ensue in this time give a second six weeks ; do not despair at the end of the second six weeks. At the end of the third six weeks do not despair. But in this case we have waited five months, and added to this there is a degree of obliquity which is unfavorable to union. At the end of this time we may despair of union taking place. The plaster of Paris dressing has been tried, but failed. Now some other means must be taken to restore the original condition and promote the formation of new material. The method suggested, of rubbing the ends of the bones together, is rarely successful, and this method, if successful, would leave the limb in its shortened condition. The practice of introducing the tenotomy knife and dividing the fibrous tissue has failed in most cases. Mr. White, of Manchester, introduced an operation which was very successful, if the patient did not die from the operation. This consisted in cutting down on the bone and taking off the fibrous ends of the fragments, and so converting the fracture into a recent compound one. This procedure was dangerous, and most of those operated upon died. In the upper limb the operation was less dangerous, but the last case I know of died of pyæmia, so that this operation was abandoned by surgeons. Then there was introduced into physics a new method of treatment, by which the danger can be avoided. What was the danger? In the simple fracture we have a layer of unbroken integument, in the compound an open wound, in which the blood and serum became putrid, and from which there oozed a stinking fluid. Cuvier introduced a piece of wood into the femoral vein of an animal, it soon became putrid. The wood containing some septic material, a similar experiment has been performed by introducing a piece of wood into the medullary canal of a bone. The animal was killed and abscess found in the liver, so we see that the bone is liable to originate a pyæmic process. Now the means of preventing this is the antiseptic treatment. By this method we now obtain better results.

A recent addition has been made to Mr. White's operation; we now drill holes through the fragments of bone and tie them together with silver wire, the wire is left in the wound, but it does not interfere with direct union ; by this operation we get a good result.

The patient was placed under the influence of chloroform. The leg was then sponged off with carbolic acid solution. An elastic tourniquet was placed around the upper part of the thigh. The carbolic spray was directed against the part of the leg where the operation was to be performed.

A superficial incision was made with the knife through the skin and superficial tissues. The extremities of the fragments were then exposed by separating the deeper structures with the periosteum detatcher. By the use of this instrument, although involving more time, there was little or no hemorrhage in the deep seated parts, which would not have been the case if the knife had been used. A piece of muscle was found to be entangled between the fragments. About one-half inch of bone was then sawed off the end of each fragment, after which, by making extension, the fragments could be approximated. A hole was then drilled through the end of each fragment, and through these openings a large silver wire was passed and firmly twisted, thus securing the close opposition of the fragments. The elastic band around the limb was then loosened and the vessels ligatured. The only vessels requiring tying were those situated in the superficial parts, where the incision had been made with the knife. The wound, about six and one-half inches in length, was then closed with silver wire, two drainage tubes being left in the wound. A piece of oiled silk protective, dipped in carbolic acid solution, was laid over the edge of the wound; over this was placed 20–40 folds of carbolized gauze, and over this the Macintosh pad. The spray was used throughout the operation, during which Mr. Lister frequently washed his hands in a solution of carbolic acid. Another important point was that the carbolic acid solution in which the instruments were kept was changed several times during the operation. Lister's long side splint was used to secure the limb in position. The splint which had been provided for the patient was too long, but Mr. Lister taking an amputating saw in a few minutes had fashioned a splint more suitable to his purpose, thus showing that his skill as a carpenter was no less than his dexterity as an operator. After the limb was dressed it was again measured. Mr. Lister stated that union would take place with only three-quarters of an inch shortening.

### WESTMINSTER HOSPITAL — SERVICE OF MR. DAVIS.

Mr. Davis, at his clinic at Westminster Hospital, presented several original methods of treatment. The first case, a middle-aged man, was admitted to the hospital fifteen months previously with a compound comminuted fracture of the leg, situated just above the malleolar. The injury was caused by a dray passing over the leg. Mr. Davis stated that it was a question whether he should amputate the foot or excise the end of the bones. In a somewhat similar case which had occurred in his practice three months ago he had excised the ends of the bone, but up to this time no union has taken place. But in this case the man had asked to have the foot amputated, and it was decided that this would be the wisest course. The wound had cicatrized but the bone had not united, and he proposed to use what he called the coat-sleeve flap, as in some former cases this had afforded him very good results. In this operation the flap is pressed together as you would the mouth of a log, a little button of cicatrix is formed in the center with lines of union radiating from it like spokes from the hub of a cart wheel; by this method he obtained a minimum amount of scar and a maximum amount of pad. After union the stump presents an appearance resembling the end of a round sofa cushion. The patient was anæsthetized and the operation performed as follows: A circular incision was made through the skin just above the malleolar, about two and one-half inches below the line of fracture, so as to allow this amount of skin to form the flap. The flap was dissected from the bone and rolled up in a manner similar to that in which we would roll up the end of a coat-sleeve. The fibrous union between the bones was then divided and the foot taken off through the fibrous tissue, thus avoiding the necessity of sawing any bone. The flap was then rolled down in its natural position and the vessels secured with hemp ligatures. No sutures were used to hold the flap in position, the edges simply being puckered together and held in position by means of a loose bandage.

A notable feature of Mr. Davis' clinic is that the spray atomizer is thrown aside to rust, and carbolic solutions and dressings are disregarded. The second case, that of a Sweedish sailor who four months previously had been thrown against the side of a boat in such a manner as to fracture the ulna a short distance below the elbow, at the same time dislocating the radius forward on to the humerus. The injury had been treated by several surgeons, but without favorable results, the arm now presenting a deformity which allowed of but limited motion of the elbow. The ulna presenting a considerable curve forward and the radius riding forward on the humerus.

Mr. Davis had decided to remove the head of the rodius, but thought it might be necessary to remove the whole joint, but would decide this question as the operation proceeded. An incision was made on the outer side of the radius and the head of the bone removed just above the tubercle; this was all that was found to be necessary. The portion of bone was removed without entering the joint, as when the dislocation occurred the head of the radius was thrown out of the joint and new adhesion had been formed. The wound was closed with wire sutures ; the limb was then supported by a piece of flexible rubber, no splint being used.

### ST. GEORGE HOSPITAL — SERVICE BY MR. PICK.

Case 1. A male patient, aged forty, was admitted to the hospital about four months ago with a compound fracture of the lower part of the leg, involving both tibia and fibula, with the ends of the bones protruding through the wound. His injury was followed by an attack of delirum tremens, this was succeeded by typhoid fever, from both of which he recovered. To these were added cellular inflammation of the injured limb, involving the foot and leg and extending nearly to the knee. After recovering from this, his general condition improved until about two weeks ago when it was discovered that necrosis had taken place in the injured bone. A portion of the diseased bone was removed, but it was found impossible to remove all. As no effort at union followed the operation it was decided to amputate. The limb was thoroughly washed with a solution of carbolic acid. A bandage was then wrapped two or three times around the thigh and over this was placed a tourniquet, the carbolic acid spray being used throughout the operation. The knife was then introduced in the front of the knee just below the patella and swept around on either side of the leg so as to form two lateral flaps. The leg was then disarticulated at the knee joint and the arteries were tied with catgut ligatures. The flaps were then approximated by silver wire, and a rubber drainage tube placed in the wound, extending up under the patella, and allowed to project at the posterior angle. A syringe was introduced between the flaps and the wound washed out with carbolic acid solution. The limb was then enveloped in a wad of gauze, consisting of twenty or thirty folds, soaked in carbolic acid solution ; over this was placed a roll of dry gauze and a piece of Macintosh, the whole being secured by a muslin roller. Mr. Pick stated that he had abandoned the operation with the long anterior flap on account of its liability to slough. He had used the lateral flaps on a former occasion and had a very good result. In this method the cicatrix formed lies in the depression between the condyles, and is thus protected from injury when the man walks.

The second patient was operated upon by Mr. Dent. Little of the history of the patient was known except that she had a disease of the knee joint which had existed for six months. It had probably originated with necrosis of the femur. The femur was now displaced backward and the deformity was continually getting worse. There was no means of straightening the limb, so that excision of the joint was the only procedure suitable to the case. After the patient was anæsthetized and the parts washed with a solution of carbolic acid, the operation was performed under the carbolic spray. A semi-circular incision was made in front of and below the patella, the flap was then dissected up and the patella removed. The diseased portion of the femur was then sawed off and the upper surface of the tibia removed and the cut surface of bone so approximated that the limb assumed the straight position. After this the wound was thoroughly syringed out ; the limb was secured with a posterior, straight splint, before the wound was closed with sutures. The wound was then approximated with silver wire sutures. A drainage tube was then secured in the wound by passing through it one of the wire sutures. Finally, the limb was bound up with carbolized gauze and a Macintosh covering. The limb was further steadied by securing it by means of two lateral splints.

Mr. Dent stated that the bones would unite, producing a stiff limb, which would still be of great service.

